# Characteristics of drug‐related deaths where individuals are found submerged in a bath or hot tub in the United Kingdom, 1997–2023

**DOI:** 10.1111/dar.13950

**Published:** 2024-09-10

**Authors:** Emmert Roberts, Caroline Copeland, Shane Darke, Michael Farrell

**Affiliations:** ^1^ National Addiction Centre, Institute of Psychiatry, Psychology and Neuroscience King's College London and the South London and the Maudsley NHS Foundation Trust London UK; ^2^ Faculty of Life Sciences and Medicine King's College London London UK; ^3^ National Drug & Alcohol Research Centre UNSW Sydney Sydney Australia

**Keywords:** bath, drowning, drug‐related deaths, epidemiology, opioids

## Abstract

**Introduction:**

Recent media reports highlight that drug‐related fatalities can occur while individuals are immersed in water in domestic settings. We aimed to determine the case characteristics, circumstances of death and type of implicated drugs among individuals dying due to unintentional drug‐related causes found immersed in a bath or hot tub.

**Methods:**

Retrospective cohort study in the United Kingdom using coronial records from the National Programme on Substance Abuse Deaths, 1997–2023. Information was available on decedent socio‐demographics, characteristics of death and drugs implicated in death.

**Results:**

One hundred fifty‐six decedents were found immersed in the bath and six in a hot tub, a mean of 6.4 deaths per year (SD 3.7; range 1–13). Overall decedents were predominantly male (*n* = 94, 58.0%), of White ethnicity (*n* = 98, 60.5%) with a mean age of 40 years (SD 13; range 19–74). Only 12 decedents had any physical contributory factor to death other than poisoning or drowning. The median number of drugs detected at post‐mortem was 3 (interquartile range 2, 5) with multiple drug toxicity implicated in the majority of cases (*n* = 90, 55.6%). The most common implicated drugs were heroin (*n* = 53, 32.7%), alcohol (*n* = 46, 28.4%) and cocaine (*n* = 33, 20.4%).

**Discussion and Conclusions:**

Over the last two decades in the United Kingdom there have been consistent numbers of unintentional drug‐related deaths each year where individuals were found in a bath or hot tub. Polysubstance, opioid and alcohol use are overrepresented. Targeted advice to avoid bathing while intoxicated would appear to be an appropriate harm reduction message.

**Key Points**

- Recent media reports highlight that unintentional drug‐related fatalities can occur while individuals are immersed in water in domestic settings.
- Over the last two decades in the United Kingdom there has been a mean of six unintentional drug‐related deaths each year where individuals were found in a bath or hot tub.
- Multiple drug toxicity was implicated in the majority of cases, with opioids implicated in over half of all fatalities.
- Targeted advice to avoid bathing while intoxicated or using multiple substances would appear to be an appropriate harm reduction message.

## 1 INTRODUCTION

A substantial number of deaths by drowning contribute to the global burden of mortality each year [1]. While a significant proportion of these deaths occur in bodies of open water, and as a result of individuals intentionally wishing to end their life [2], recent media reports have highlighted that unintentional fatalities can occur while individuals are immersed in water in domestic settings [3].

In the United Kingdom the number of unintentional deaths by drowning is estimated to be approximately 400 per year [4], with the number of drug‐related death registrations reaching a record high of 6112 in 2022 [5, 6, 7]. Although drug and/or alcohol use has been consistently reported to be a contributory cause of death in many intentional and open water drownings [1], there has, to our knowledge, been limited systematic exploration of the role of specific drug and/or alcohol toxicity where people are found immersed in water in domestic settings [8, 9]. Given the nature of these deaths, if specific case characteristics and/or particular drug use profiles were found to be associated with these fatalities this could lead to the development of targeted interventions or specific harm reduction advice. Several studies also note significant differences in the types of drugs implicated in drug‐related death when decedents are stratified by sex, though, to our knowledge, this has also not been explored in this context [10].

To address these gaps, we aimed to determine: (i) the case characteristics; (ii) the circumstances of death; and (iii) the number and type of drugs implicated in death among people dying due to unintentional drug‐related causes who were found immersed in water in either a bath or hot tub in the United Kingdom. We also aimed to assess any differences stratified by sex.

## 2 METHODS

This study is reported according to the Strengthening the Reporting of Observational Studies in Epidemiology (STROBE) statement [11], the completed checklist available as Table S1, Supporting Information.

### 2.1 The national programme on substance abuse deaths

We conducted a retrospective cohort study using records drawn from the National Programme on Substance Abuse Deaths (NPSAD) [10, 12]. This database contains information on an observational cohort that collects coronial data on deaths at any age related to any psychoactive drug, other than nicotine, caffeine or where the sole substance implicated is alcohol [13]. In total, NPSAD holds records on more than 50,000 deaths with reports received from England, Wales, the Channel Islands and the Isle of Man since 1997. Additional reports were received from the Scottish Crime and Drug Enforcement Agency between 2004 and 2011 and from the General Register Office for Northern Ireland since 2004. Deaths are referred to a Coroner if the cause of death is unknown, is violent or unnatural, sudden and unexplained, occurred during or following a period of anaesthesia or may have been caused by an industrial disease or poisoning [14]. Coronial files typically include the coroner's decision as to cause of death, statements from witnesses, family and friends, first responders (e.g., police, emergency services), general practitioner records and hospital records. Toxicology is discretionally requested by individual Coroners and Coronial jurisdictions choose to voluntarily report all deaths within their area to NPSAD if any of the following criteria are met: (i) psychoactive substance(s) are directly implicated in death; (ii) an individual has a history of dependence or misuse of drugs; or (iii) if controlled drugs are identified at post‐mortem. The King's College London Biomedical and Health Sciences, Dentistry, Medicine and Natural and Mathematical Sciences Research Ethics Subcommittee re‐confirmed in October 2023 that NPSAD does not require Research Ethics Committee review as all individuals are deceased.

### 2.2 Case identification

All cases where the cause of death was deemed unintentional by the Coroner (i.e., neither a suicide nor a homicide) between 1997 and April 2023 were identified. NPSAD fields were subsequently searched for the following terms: ‘bath*’, ‘*tub*’ and ‘jacuzzi*’. Authors manually reviewed the text of Coronial files to confirm each decedent was found submerged in water in either a bath or hot tub with final searches conducted on 23 January 2024.

### 2.3 Measures

Data on decedent socio‐demographics, mental health history (i.e., a documented history of individual mental health conditions), substance use history (i.e., documented history of substance dependence and injecting status) and which individual substances were ultimately deemed implicated in death were extracted from Coronial files. All cases additionally recorded the findings concerning the disease or condition that led directly to death, any antecedent cause(s) and other significant conditions associated with death.

### 2.4 Statistical analysis

We initially generated descriptive statistics for all decedents stratified by sex. Means, standard deviations (SDs) and ranges were presented for continuous variables, as well as median and interquartile range in skewed distributions. Percentages were reported for binary and categorical variables. Continuous variables were compared using an unpaired *t*‐test, ordinal variables using the Wilcoxon rank sum test and categorical variables using a chi‐squared test. Where cell sizes were five or less categorical variables were compared using Fisher's exact test [15]. All analyses were conducted in STATA IC version 15, with the significance level set at 0.05. The analysis plan was not pre‐registered and the results should be considered exploratory.

## 3 RESULTS

A total of 156 decedents were found immersed in the bath and six in a hot tub. There was a mean of 6.4 deaths per year (SD 3.7; range 1–13). This is compared to a mean of 1919 deaths per year (SD 438.2; range 527–2646) within the entire NPSAD database over a contemporaneous timeframe.

### 3.1 Socio‐demographics and case characteristics

Socio‐demographic and case characteristics of all decedents stratified by sex can be found in Table 1. Overall decedents were predominantly male (*n* = 94, 58.0%), of White ethnicity (*n* = 98, 60.5%) and had a mean age of 40 years (SD 13; range 19–74). A substantial proportion of all decedents had no recorded ethnicity (*n* = 58, 35.8%) and were unemployed at the time of their death (*n* = 63, 38.9%). Almost three‐quarters of decedents died while in their own accommodation (*n* = 120, 74.1%), the remainder having died in another private residence (*n* = 11, 6.8%), a hotel (*n* = 5, 3.1%) or died in hospital having been subsequently conveyed following initial discovery immersed in a bath or hot tub in a domestic setting (*n* = 24, 14.8%). When comparing men to women there was no clear evidence of any difference in socio‐demographics by sex.

**TABLE 1:** Socio‐demographic and case characteristics of people dying due to unintentional drug‐related causes found immersed in water in either a bath or hot tub in the United Kingdom, 1997–2023.

	All, *n* = 162	Males, *n* = 94	Females, *n* = 68
	*n* (%)	*n* (%)	*n* (%)
Age			
Mean years	40.4 (SD 13.0; range 19–74)	39.2 (SD 12.0; range 19–72)	42.1 (SD 14.2; range 19–74)
Ethnicity			
White	98 (60.5)	56 (59.6)	42 (61.8)
Black Caribbean	1 (0.6)	0 (0.0)	1 (1.5)
Pakistani	1 (0.6)	1 (1.1)	0 (0.0)
Other	4 (2.5)	2 (2.1)	2 (2.9)
Unknown/not recorded	58 (35.8)	35 (37.2)	23 (33.8)
Occupation^a^			
Employed (manual)	32 (19.8)	28 (29.8)	4 (5.9)
Employed (non‐manual)	9 (5.6)	4 (4.3)	5 (7.4)
Self‐employed	8 (4.9)	5 (5.4)	3 (4.4)
Childcare/house person	3 (1.9)	0 (0.0)	3 (4.4)
Student	5 (3.1)	3 (3.2)	2 (2.9)
Unemployed	63 (38.9)	32 (34.0)	31 (45.6)
Retired	12 (7.4)	5 (5.3)	7 (10.3)
Long‐term sickness	6 (3.7)	6 (6.4)	0 (0.0)
Unknown	24 (14.8)	11 (11.7)	13 (19.1)
Year of death			
1999–2003	18 (11.1)	12 (12.8)	6 (8.8)
2004–2008	40 (24.7)	20 (21.3)	20 (29.4)
2009–2013	29 (17.9)	17 (18.1)	12 (17.7)
2014–2018	34 (21.0)	17 (18.1)	17 (25.0)
2019–2023	41 (25.3)	28 (29.8)	13 (19.2)
Place of death			
Own place of residence	120 (74.1)	67 (71.3)	53 (77.9)
Other residential address	11 (6.8)	7 (7.4)	4 (5.9)
Hotel	5 (3.1)	4 (4.3)	1 (1.5)
Hospital	24 (14.8)	16 (17.0)	8 (11.8)
Not reported	2 (1.2)	0 (0.0)	2 (2.9)

Abbreviation: SD, standard deviation.

^a^ Occupation definitions are based on those used by the United Kingdom Office of National Statistics [16].

### 3.2 Characteristics of death and mental health and substance use history

Circumstances of death and mental health and substance use histories of all decedents stratified by sex can be found in Table 2. The most common diseases or conditions directly leading to death were accidental poisonings (*n* = 59, 44.7%; International Classification of Diseases Version 10 [ICD‐10] codes X40–X45), poisonings of undetermined intent (*n* = 11, 6.8%; ICD‐10 codes Y10–Y14) and accidental drowning or submersion (*n* = 28, 17.3%; ICD‐10 codes W65–W74) with only single digit deaths either directly caused by, or having antecedents related to, cardiovascular, respiratory or gastrointestinal pathology. Only 12 decedents had physical diseases or conditions recorded on their death certificate which were deemed contributory to the occurrence of the death but were not part of the main sequence leading to death. These included ischaemic heart disease, chronic viral hepatitis, myocarditis, acute liver injury and congenital malformations. There was no clear evidence of any difference in the causes of death by sex.

**TABLE 2:** Circumstances of death and mental health and substance use history among people dying due to unintentional drug‐related causes found immersed in water in either a bath or hot tub in the United Kingdom, 1997–2023.

	All, *n* = 162	Males, *n* = 94	Females, *n* = 68
	*n* (%)	*n* (%)	*n* (%)
Direct cause of death (ICD‐10 code)			
Poisoning, accidental (X40–X45)	59 (44.7)	37 (39.4)	22 (3.4)
Poisoning, undetermined intent (Y10–Y14)	11 (6.8)	6 (6.4)	5 (7.4)
Accidental drowning and submersion (W65–W74)	28 (17.3)	12 (12.8)	16 (23.5)
Heroin overdose (T40.1)	1 (0.6)	0 (0.0)	1 (1.5)
Cocaine overdose (T40.5)	2 (1.2)	1 (1.1)	1 (1.5)
Cardiac arrythmia (I49.9)	2 (1.2)	1 (1.1)	1 (1.5)
Cardiac arrest, unspecified (I46.9)	1 (0.6)	0 (0.0)	1 (1.5)
Brain damage, anoxic or hypoxic (G93.1)	1 (0.6)	1 (1.1)	0 (0.0)
Adult respiratory distress syndrome (J80)	1 (0.6)	1 (1.1)	0 (0.0)
Aspiration pneumonia (J69.0)	1 (0.6)	1 (1.1)	0 (0.0)
Haemorrhage, not elsewhere classified (R58)	1 (0.6)	1 (1.1)	0 (0.0)
Unascertained (R99)/not reported	54 (33.3)	33 (35.1)	21 (30.9)
Mental health			
People with a history of any mental health issue	53 (32.7)	31 (33.0)	22 (32.4)
People with a history of schizophrenia	9 (5.6)	6 (6.4)	3 (4.4)
People with a history of bipolar affective disorder	9 (5.6)	4 (4.3)	5 (7.4)
People with a history of a depressive disorder	37 (22.8)	21 (22.3)	16 (23.5)
People with a history of an anxiety disorder	17 (10.5)	10 (10.6)	7 (10.3)
People with a history of PTSD	3 (1.9)	2 (2.1)	1 (1.5)
Addiction			
People with a history of substance dependence	94 (58.2)	58 (61.7)	36 (52.9)
People with an injecting history	15 (9.3)	9 (9.6)	6 (8.8)

Abbreviations: ICD‐10 International Classification of Diseases Volume 10; PTSD post‐traumatic stress disorder; SD standard deviation.

In cases where medical history was available, almost a third of all decedents had a history of any mental health condition (*n* = 53, 32.7%), the two most common documented mental health conditions being a depressive disorder or an anxiety disorder. The majority of decedents had a history of substance dependence (*n* = 94, 58.2%), with almost 10% of decedents having a history of injecting (*n* = 15, 9.3%). A significantly higher proportion of males had a history of substance dependence (23.4% vs. 13.2%, *p* = 0.012) and there was no clear evidence of any difference in any other mental health or substance use history by sex.

### 3.3 Number and type of drugs implicated in death

The number and type of drugs implicated in death of all decedents stratified by sex can be found in Table 3. The median number of drugs detected at post‐mortem was 3 (interquartile range 2, 5) with multiple drug toxicity implicated in the majority of cases (*n* = 90, 55.6%). The three most common drug groups implicated in death were opioids, benzodiazepines and gabapentinoids, with the three most common individual drugs implicated being heroin, alcohol and cocaine. A minority of decedents had a drug for which they had a prescription implicated in death (*n* = 41, 25.3%), the three most common prescribed drugs being diazepam, methadone and zopiclone.

**TABLE 3:** The number and type of drugs implicated in death among people dying due to unintentional drug‐related causes found immersed in water in either a bath or hot tub in the United Kingdom, 1997–2023.

Drug implicated in death^a^		All, *n* = 162	Males, *n* = 94	Females, *n* = 68
		*n* (%)	*n* (%)	*n* (%)
All	Mean number of drugs implicated	3.7 (SD 2.0; range 1–14)	3.7 (SD 2.3; range 1–14)	3.7 (SD 1.7; range 1–8)
	Median number of drugs implicated	3 (IQR 2, 5)	3 (IQR 2, 5)	4 (IQR 2, 5)
	Only single substance implicated	72 (44.4)	42 (44.7)	30 (44.1)
	Multiple substances implicated	90 (55.6)	52 (55.3)	38 (55.9)
Prescribed	Any prescribed drug implicated	41 (25.3)	22 (23.4)	19 (27.9)
Opioids	Any opioid	83 (51.2)	53 (56.4)	30 (44.1)
	Heroin	53 (32.7)	34 (36.2)	19 (27.9)
	Methadone	12 (7.4)	6 (6.4)	6 (8.8)
Benzodiazepines	Any benzodiazepine	56 (34.6)	29 (30.9)	27 (42.2)
	Diazepam	18 (11.1)	6 (6.4)	10 (14.7)
Z Drugs^b^	Any Z Drug	14 (8.6)	3 (3.2)	11 (16.2)
	Zopiclone	13 (8.0)	3 (3.2)	10 (14.7)
Gabapentinoids	Any gabapentinoid	16 (9.9)	12 (12.8)	4 (5.9)
Amphetamines	Any amphetamine	11 (6.8)	10 (9.4)	1 (1.5)
Alcohol	Alcohol^c^	46 (28.4)	24 (25.5)	22 (32.4)
Cocaine	Cocaine	33 (20.4)	23 (24.5)	10 (14.7)
Ketamine	Ketamine	9 (5.6)	5 (5.3)	4 (5.9)

Abbreviations: IQR, interquartile range; SD, standard deviation.

^a^ Only those substances implicated in five or more deaths are reported.

^b^ Zopiclone, eszopiclone, zaleplon or zolpidem.

^c^ The Nature of the National Programme on Substance Abuse Deaths database is such that deaths in which alcohol is the only substance implicated are not recorded. As such drug‐related deaths in which alcohol is implicated in National Programme on Substance Abuse Deaths have, by definition, multiple substances implicated.

While there was no clear evidence of any difference by sex in the number of drugs detected at post‐mortem, or the proportion of decedents with multiple drug toxicity implicated in death, in males there was a significantly higher proportion of deaths in which any amphetamine was implicated (9.4% vs. 1.5%, *p* = 0.02) and a significantly lower proportion in which any Z drug (i.e., zopiclone, eszopiclone, zaleplon or zolpidem) (3.2% vs. 16.2%, *p* = 0.004) or zopiclone (3.2% vs. 14.7%, *p* = 0.008) was implicated.

## 4 DISCUSSION

Over the last two decades within the NPSAD cohort there has been a mean of six unintentional drug‐related deaths per year where individuals were found submerged in a bath or hot tub. Multiple drug toxicity was implicated in the majority of cases, with opioids implicated in over half of all fatalities. Indeed, the case characteristics were typical of UK drug‐related deaths with decedents being majority male, having an average age of 40, with a high proportion unemployed, and having a history of substance dependence [17]. There were, however, considerably fewer decedents with physical conditions or co‐morbidities that contributed to death when compared to the overall population of individuals dying due to drug‐related causes in the United Kingdom, with fewer than 10% of the decedents studied having a death certificate record of any cause or contributing factor other than poisoning or drowning.

The preponderance of opioids, and in particular heroin, is notable in that prevailing understanding of opioid overdose would suggest that the act of submersion in water, and the subsequent stimulus and shock of ingestion of water, while intoxicated could trigger individuals to breathe and prevent drowning [18]. Additionally, fewer than 10% of decedents had a history of injecting suggesting the majority of opioid consumption implicated in deaths was via other routes. Previous research has established that opioid consumption in bathrooms is common, largely due to reasons of privacy, the security of a locked door and availability of water [19, 20]. While harm reduction advice would routinely encourage people who use opioids to avoid using alone, and to carry take‐home naloxone, advice of avoiding immersion in water while intoxicated is not commonplace, nor is the specific suggestion of storing a take‐home naloxone kit in the bathroom. Unlike deaths that occur in open water, a unique feature of fatalities occurring in a bath or hot tub is that the water in question is often heated. This has the potential to alter physiological processes with possible implications on the pharmacokinetic profile of any drugs consumed, either individually or in combination, and could be a contributory factor to increased risk of toxicity [21]. Given the majority of fatalities involved multiple drug toxicity, and a substantial number implicated sedative substances, avoidance of polysubstance use prior to bathing would also appear to be an important message. There were limited differences when stratified by sex other than a greater proportion of women having Z drugs implicated in death, this observation likely reflective of sex differences in prescribing in the United Kingdom [22].

The study has several strengths including the granular examination of substances implicated in death and the large time frame. There are, however, several important limitations. Within NPSAD potential variables of interest may not be available or their quality and completion inconsistent across coronial jurisdictions. There are significant proportions of incomplete reporting for some variables (e.g., over a third of cases contain no reported ethnicity), which limits the ability to comment on key factors that may expose significant inequities. NPSAD also does not contain information on alcohol‐only deaths or on which decedents were accessing evidenced‐based harm reduction or addiction treatment, thus knowledge of the proportion of people to whom specific harm reduction advice could be delivered in these settings is unknown. Future data linkage to treatment records may thus be a fruitful avenue for further study. In all studies of mortality, determination of intentionality is subjective. A further inherent limitation in studies using coronial records is that, consideration of intentionality derives from individual coronial conclusions and thus a number of deaths may have been misclassified. Additionally, due to the voluntarily submission nature, NPSAD has incomplete coverage of the UK coronial system over the studied timeframe, with variability of toxicological examination in all cases, thus some cases will likely have been missed, and some implicated substances not identified. These are likely to lead to an underestimation in both the number of decedents and implicated drugs. However, the study does remain, to our knowledge, the largest epidemiological examination of drug‐related deaths where decedents are found immersed in water in domestic settings.

In summary, there have been consistent numbers of drug‐related deaths where decedents are found immersed in water in a bath or hot tub over the past two decades. Polysubstance, opioid and alcohol use are overrepresented and targeted harm reduction advice to avoid bathing while intoxicated or using multiple substances would appear to be an appropriate message directed towards people who use drugs and those caring for them.

## AUTHOR CONTRIBUTIONS

Each author certifies that their contribution to this work meets the standards of the International Committee of Medical Journal Editors.

## CONFLICT OF INTEREST STATEMENT

All Individual authors have completed the ICJME conflict of interest form. The authors have no interests to declare.

## Supporting information


**Table S1.** Supporting information.

## Data Availability

Data are available subject to institutional approvals.
